# 肺癌类器官的规范化构建及其在肺癌精准诊疗中的应用进展

**DOI:** 10.3779/j.issn.1009-3419.2026.102.20

**Published:** 2026-06-20

**Authors:** Sen CAO, Wen LI, Xiaokuan ZHANG, Tianyi NIU, Zheng WU, Zhiyu WANG

**Affiliations:** ^1^050011 石家庄，河北医科大学第四医院肿瘤免疫科（曹森，张晓宽，牛天翼，吴峥，汪治宇）; ^1^Department of Immuno-Oncology, The Fourth Hospital of Hebei Medical University, Shijiazhuang 050011, China; ^2^050017 石家庄，河北医科大学肿瘤学系（李文）; ^2^Department of Oncology, Hebei Medical University, Shijiazhuang 050017, China

**Keywords:** 肺肿瘤, 肺癌类器官, 药物敏感性检测, 肿瘤微环境, Lung neoplasms, Lung cancer organoids, Drug sensitivity testing, Tumor microenvironment

## Abstract

肺癌是我国发病风险最高、死亡率最高的癌症类型。尽管随着多种新型治疗手段的应用，肺癌的治疗较前已经取得显著进展。然而肺癌早期诊断困难的问题仍然存在，超过半数患者在诊断时已经发生了远处转移，这与理想中的精准治疗仍存在较大的差距。因此迫切需要开发一种新型的个体化诊疗模型。传统的癌细胞系模型缺乏空间结构，无法真实还原亲本组织的生长变化；患者来源异种移植模型造价昂贵，移植成功率低。肺癌类器官（lung cancer organoids, LCOs）模型相较于其他模型稳定遗传性高，培养迅速，不仅可真实模拟原始肿瘤的自然生长变化，而且减少了对动物的依赖，更加适合大规模药物筛选。然而随着类器官技术的快速发展，LCOs的构建方法尚未达成一致意见，本文系统阐述了LCOs模型的标准化构建及其在肺癌精准诊疗中的应用和最新进展情况，以期指导其临床应用，进而提高患者的生存质量，延长生存期。

## Abstract (en)

Lung cancer is the malignancy with the highest incidence and mortality rates in China. Although significant progress has been made in its treatment due to the application of various novel therapeutic approaches, challenges remain in early diagnosis, as more than half of patients present with distant metastases at diagnosis, indicating a substantial gap compared to ideal precision therapy. Therefore, there is an urgent need to develop a novel personalized diagnostic and therapeutic model. Traditional cancer cell line models lack spatial structure and fail to accurately replicate the growth dynamics of native tissues; patient-derived xenograft models are costly and exhibit low transplantation success rates. In contrast, lung cancer organoids (LCOs) demonstrate superior genetic stability and rapid culture efficiency compared to other models, faithfully simulating the natural growth patterns of primary tumors and reducing animal dependency, making them particularly suitable for large-scale drug screening. However, despite rapid advancements in organoid technology, consensus has yet to be reached regarding standardized LCOs construction methods. This article systematically outlines the standardized development of LCOs models, their applications in precision lung cancer diagnosis and treatment, and recent progress, aiming to guide clinical implementation and improve patients' quality of life and survival outcomes.

肺癌已经成为全球发病率最高、经济负担最重、死亡威胁最大的癌症类型^[1]^。非小细胞肺癌（non-small cell lung cancer, NSCLC）是肺癌最主要的病理亚型，占85%-88%，小细胞肺癌（small cell lung cancer, SCLC）仅占12%-15%。随着影像学检查手段更加精准化，分子靶向治疗（molecular targeted therapy, MTT）和免疫治疗等新型治疗手段逐渐应用，癌症的诊断与治疗较前已经取得十足进展，这在NSCLC中尤为显著^[2,3]^。但早期诊断困难仍然是一个重要问题，超过半数患者一经确诊即已发生远处转移。由于肿瘤异质性和基因组不稳定性的存在常导致耐药机制的产生^[4]^。这与理想中的精准治疗仍存在较大差距。因此迫切需要开发一种能够准确反映肿瘤生物学特性的体外模型，以提高肺癌患者的生存质量，延长生存期。

癌细胞系（cancer cell line, CCL）模型利用患者来源的肿瘤细胞进行培养，培养周期短，花费低，但基因组稳定性差，并且在长期培养过程中会导致积累遗传改变，无法真实还原原始肺癌细胞的生长变化^[5,6]^。来源肿瘤异种移植（patient-derived xenografts, PDXs）模型能够保持原始肿瘤组织病理学结构和基因表达谱，但易受动物免疫微环境的影响，稳定性较差^[6,7]^；且价格昂贵，移植成功率低^[8]^。肺癌类器官（lung cancer organoids, LCOs）模型兼具两者优势，培养周期短、成功率高、新鲜的肿瘤细胞或组织、恶性渗出液、循环肿瘤细胞（circulating tumor cells, CTCs）都可成为其培养成功的原材料。同时LCOs模型的基因的遗传稳定性也更强，在体内外均能真实地模拟原始肿瘤的自然生长变化^[9]^，避免了动物本身肿瘤特异性进化对原始肿瘤的影响^[10]^。随着LCOs的快速发展和进步，关于其构建方法层出不穷，应用范围也越来越广泛。但是利用LCOs进行机制探索和用于药物敏感性检测仍处于起步阶段。本文将对LCOs的构建及其在肺癌精准治疗模式中的应用进行系统综述，为推动标准化LCOs模型的构建和临床转化应用提供指导，以期肺癌的精准治疗能够早日实现。

## 1 LCOs概述

类器官模型跨越生物工程、临床医学与干细胞培养等多学科进行技术交叉融合，首次在体外环境下模拟器官的复杂结构与功能，为生命科学开辟了全新的研究范式。肿瘤类器官模型可以在体外甚至免疫缺陷异种移植小鼠内精准保留原始肿瘤组织的病理生理特征和遗传异质性，且能够长期稳定地进行培养和传代^[11]^。但不同组织来源肿瘤类器官构建成功率大不相同，非上皮组织干细胞稀少，缺乏自组织倾向，往往需要复杂的体外刺激才能模拟真实器官功能；上皮组织来源类器官干细胞含量高，具有高度自组织性，能更真实地模拟复杂的肿瘤微环境（tumor microenvironment, TME）和上皮细胞免疫细胞相互作用^[12]^，构建成功率也更高（47%-85%）^[13,14]^，临床转化潜力巨大。科研人员已成功构建出结直肠癌^[15]^、乳腺癌^[16]^、肺癌^[17]^等多种上皮组织来源肿瘤类器官，并且成功利用这些模型用于探索肿瘤发病机制、患者用药反应以及新型抗癌药物的研发等方面的研究^[18]^。

肺癌作为全球发病率及死亡率最高的癌症类型，LCOs模型的构建和临床转化应用在类器官发展简史中有着举足轻重的地位，为探究疾病机制、阐明分子通路及探索新型治疗靶点提供了卓越的研究平台。然而不同实验室构建LCOs的流程缺乏统一标准，构建成功率存在显著差异^[19]^。同时由于缺乏免疫细胞和成纤维细胞等TME成分，LCOs的模拟能力并不十分精确。因此规范LCOs模型的构建流程始终是LCOs转化研究领域一道重要难题。

## 2 LCOs的规范化构建

LCOs的构建涉及多重复杂的步骤和条件，从亲本组织的筛选、取样条件、基质胶的选择和构建成功的评价标准等方面均需进行严格把控，以促使不同研究团体之间制定出统一的构建标准，提高LCOs模型构建成功率，为有效开展后续临床转化研究工作保驾护航。

### 2.1 LCOs的样本来源、培养流程和评价标准

肺癌手术切除标本是最常用的样本。除此之外研究者们还广泛应用活检组织、恶性积液、冻存组织和CTCs等标本进行LCOs模型的构建^[20-24]^。收集标本的同时也要注意完整细致地记录患者的标本状态、肿瘤信息和精准的临床信息以便基础转化实验的有效开展。收集好的组织样本应保存在装有类器官专用保存液的无菌移液管中，于4 ^o^C低温环境下快速转运至实验室以尽量保持细胞活性状态。并于72 h内进行清洗和机械消化，通过细胞筛网过滤收集细胞悬液。

基质胶不仅是维持类器官模型3D空间结构的“支架”，更是类器官形成和分化必不可少的成分。水凝胶是现阶段LCOs培养最为常用的基质胶类型，然而由于其成分复杂，易出现信息失真，临床转化意义十分有限。为此Wang等^[25]^通过将来源于肿瘤组织碎片的中尺度胶原蛋白束和微生物转谷氨酰胺酶交联明胶相结合，成功构建了一种名为“MC-gel”的复合水凝胶，与常用的基质胶相比，在MC-gel中生长的LCOs表现出更大直径、更高细胞活力和更接近体内观察到的形状。相较于传统水凝胶结构，MC-gel在研究肿瘤的发生转移进展和模拟患者治疗结局方面表现出更大的潜力，可有效整合至肿瘤的个性化治疗方案中^[26]^。培养基是LCOs培养成功的关键决定因素，应根据不同的培养目的选择最适宜的培养基。有研究^[27]^结果表明LCOs在有限培养基中培养成功率较高，而在允许培养基和最小基础培养基中状态较差。

为保证所构建的LCOs模型与亲本组织之间的组织特性一致，应定期通过光学显微镜和苏木素-伊红（hematoxylin eosin, HE）染色观察类器官的形态特征，确定其结构是否与原始肿瘤相似。通过实时荧光定量聚合酶链式反应（quantitative real-time polymerase chain reaction, RT-qPCR）或RNA测序技术评估肺癌相关基因表达情况，如检测表皮生长因子受体（epidermal growth factor receptor, *EGFR*）、Kirsten大鼠肉瘤病毒癌基因同源物（Kirsten rat sarcoma viral oncogene homolog, *KRAS*）、*TP53*等突变基因的表达水平，验证类器官的肿瘤特性。通过免疫组化染色检测特定肿瘤标志物以进一步确认类器官是否具有肺癌细胞的表型。全基因组测序和质谱技术分析类器官的基因组变异和蛋白质表达谱，进一步确认其与临床样本的一致性。经上述鉴定合格的类器官可进行后续基础研究和临床转化工作^[28,29]^（图1）。

**图 1:**
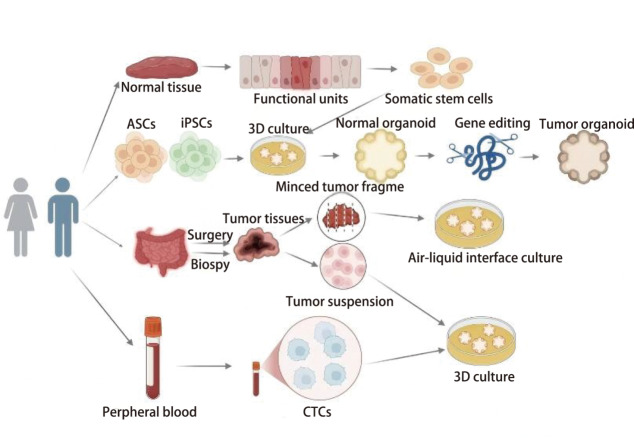
LCO的标准化构建流程图

### 2.2 LCOs多维度量化质控体系的建立

由于缺乏统一、可量化的质控标准，不同实验室构建LCOs的成功率存在显著差异（30%-80%）^[19,30]^。为明确肿瘤类器官构建的统一技术规范，提高实验结果的可重复性，近年来国内外专家提出建立覆盖多维度的量化评价体系，统一判定阈值以提升实验的可重复性与临床转化价值^[11]^。

新鲜的肿瘤组织样本经消化过滤后应即刻评估细胞活性，细胞存活率≥70%可选择接种于低温孔板中进行培养，若细胞活性≥90%则为最优选择组织来源。采用恶性胸腹水、转移组织、CTCs等进行LCOs模型的构建前应进行密度梯度离心以尽量去除杂细胞，提高构建成功率。细胞接种成功后每隔2-3天应定期更换1次培养基，并注意观察类器官的数量和形态变化，以免因营养缺乏、过度拥挤而造成细胞死亡。通常培养1周即可初步在显微镜下观察到类器官结构，此时应注重观察有无污染，营养物质是否充足。培养2-3周后类器官直径超过300 µm，表明类器官生长进入平台期，此时可考虑进行传代扩增或进行后续实验。

在每个类器官系成功建立的P0代、进行关键研究或冻存入生物样本库的P3代、P5代均应进行组织病理学、免疫组化和基因组测序以对该模型进行验证。肿瘤类器官建立的初衷即能够在体外真实地再现患者的基因遗传特征，从而降低或减少药物不良反应的发生，采用多层策略和方法全面评估模型的保真度是我们质量控制的核心目标^[31]^。

### 2.3 LCO TME的构建

TME是肿瘤细胞赖以生存的“土壤”，它不仅为肿瘤生长提供所需的营养物质，还参与调控肿瘤的增殖、侵袭和转移等关键过程。肿瘤免疫微环境（tumor immune microenvironment, TIME）则更加复杂，是一种涉及多种细胞类型、信号通路及其相互作用的动态生态系统，不仅影响肿瘤的生长、转移和对治疗的反应，还特异性地调控抗肿瘤免疫应答，决定肿瘤免疫逃逸机制^[32]^。

复杂的TME和患者的基因遗传特征是影响患者抗肿瘤药物治疗反应的重要条件^[33]^。现有模型仍不足以完全复刻肿瘤在体内进展情况，进而导致其结果缺乏可靠性与真实性。因此亟须建立一种既能够精准复现肺癌患者的体内遗传特性，又能完整构建TIME特征的体外癌症模型，以更精确地评估药物疗效。对此Choi等^[34]^利用3D生物打印技术成功构建了具有基质细胞、细胞外基质成分和灌注血管结构的先进肺泡细胞模型。以此培养的LCOs模型不仅保留了肺腺癌的亚型特性和基因突变特征，而且生长迅速，该模型有望应用于新的生物标志物的发现以及评估新药物疗效。患者来源类器官（patient-derived organoids, PDOs）模型和自体免疫细胞共培养被认为是能够在体内重建体内TME的重要技术^[35,36]^。利用该互作体系不仅可研究肿瘤免疫逃逸机制，评估免疫治疗疗效反应，还可用于开发能够增强免疫疗效的新型药物。Gao等^[37]^提出了量化LCOs模型和自体免疫细胞之间相互作用的微流体芯片，“OI-Chip”量化了类器官模型和自体免疫细胞间的两个水平相互作用、免疫介导的杀伤行为和多种免疫细胞因子的能力，为患者免疫反应的离体评估奠定了基础。未来的发展方向主要包括提高模型的标准化和长期稳定性以增强模型的预测能力，加速免疫细胞疗法的临床转化。

免疫治疗是利用TME对抗肿瘤的免疫逃逸，增强人体自身免疫功能的治疗手段。然而面对存在复杂TIME的实体肿瘤患者，可从免疫治疗中获益的人群范围越来越小。为此Wisdom等^[38]^开发了一种能够在肺癌中促进T细胞浸润的芯片模型，该模型可以模拟T细胞黏附、外渗和迁移过程，评估免疫趋化性对肿瘤的增强作用，同时探测血管对潜在治疗的反应。

## 3 LCOs在肿瘤精准治疗中的应用

### 3.1 探索肺癌发生发展机制

肺癌发生发展的机制至今尚未完全阐明。YES相关蛋白（Yes-associated protein, YAP）是一种常见的致癌蛋白，与多种恶性肿瘤的进展显著关联^[39,40]^，YAP-SAV1作为肺上皮细胞稳态维持的重要负反馈通路，在建立YAP调控的稳健性和稳态机制方面具有重要作用。Liu等^[41]^利用LCOs模型发现联合甲基化抑制剂和YAP-TEAD抑制剂为肺癌患者提供了一种全新的基因治疗的潜在靶点。SCLC模型的研究与开发是目前临床治疗的难点。Sen等^[42]^利用功能化的藻酸盐微珠模拟肺泡结构成功开发出了一种3D SCLC共培养类器官模型，通过与患者原代肿瘤细胞对比发现它们不仅构建成功率高，还可有效保留患者的基因和遗传突变特征。

癌细胞通常需要进行复杂的代谢重编辑来补给能量，以适应侵袭转移过程中的恶劣环境，其中脂质代谢对于提供各类脂质作为燃料或信号分子以满足其增长和信号转导的高需求至关重要。胆固醇作为最主要的脂质种类之一，在癌症的发生进展过程中既可以直接促进癌症的发生进展，也可以抑制癌细胞的转移^[43,44]^。但肺部胆固醇如何积聚、如何影响肺癌发展的调控机制至今仍未完全阐明。叉头框蛋白A3（forkhead box protein A3, FOXA3）是调控体内脂质代谢及葡萄糖代谢的关键转录因子，参与肺部生理病理过程。Wang等^[45]^利用LCOs模型发现外源性胆固醇作为转移性肺腺癌的关键信号分子，可以通过GLI2/FOXA3/HMGCS1轴激活胆固醇从头生物合成，促进膜组成变化，进而增强肺癌的远处转移能力。在肺腺癌中*FOXA3*的敲低或敲除可以有效抑制转移相关基因程序，由此可见在肺腺癌中抑制胆固醇从头合成途径的重要性，也为癌症患者的营养干预提供了新的治疗策略。

### 3.2 新型治疗靶点的探索

奥希替尼作为第三代EGFR-酪氨酸激酶抑制剂（EGFR-tyrosine kinase inhibitors, EGFR-TKIs）临床疗效显著，但对NSCLC患者的治疗效果最终受限于获得性耐药的出现，超过半数患者的耐药机制至今尚未明确。探索并阐明肺癌的发病规律和耐药机制，对于改善当前肺癌的诊疗环境至关重要。肿瘤时空异质性是肺癌靶向治疗原发耐药与治疗后复发的核心根源。类器官保留了原始组织特有的基因组变异、分子特征和异质性，同时微流控芯片和组织工程技术的应用也有助于实现多点微量样本体外建系。通过建立药物耐药和药物敏感的类器官模型解析肿瘤时空异质性、探索新型治疗靶点和耐药机制具有潜在价值，在加深我们对疾病认识的同时也有助于改善目前的诊疗环境。

现有研究借助上述演化模型证实，长链非编码RNA（long noncoding RNA, lncRNA）和YAP是肿瘤的生长增殖、凋亡逃逸等过程的关键调节因子，在靶向药物耐药性出现中起核心作用^[46]^。富含亮氨酸重复序列的卵巢肿瘤抑制因子（leucine-rich repeat-containing ovarian tumor suppressor, LRTOR）不仅能够促进肿瘤生长，而且能够引发K63连接的多聚泛素化修饰，建立起放大YAP致癌信号的正反馈循环，最终赋予奥希替尼耐药性。Miao等^[47]^利用该机制成功鉴定出一种新型lncRNA——LRTOR，揭示了LRTOR是奥希替尼耐药的核心调控因子，并且可作为克服*EGFR*突变NSCLC患者奥希替尼耐药后的新型治疗靶点与预后标志物。

RNA剪接失调已成为癌症的普遍特征，Ras相关C3肉毒梭菌毒素1B（Ras-related C3 botulinum toxin substrate 1B, RAC1B）通过与鸟嘌呤核苷酸交换因子GDS1特异性结合介导自身激活来抑制EGFR的溶酶体运输和降解稳定EGFR。然而RAC1B和RAC1A等主要RAC1异构体在癌症中的治疗意义仍未得到探索。Yan等^[48]^通过利用肺腺癌的PDOs模型，发现靶向RAC1B可以有效抑制肿瘤生长并增强EGFR-TKIs治疗功效，强调了一种在肺腺癌中有前途的新型靶向治疗途径。Ji等^[49]^研究发现NRF2信号下调抑制了肺鳞癌细胞系和类器官的增殖，此外还会降低磷脂酰肌醇3-激酶/哺乳动物雷帕霉素靶蛋白（phosphoinositide 3-kinase/mammalian target of rapamycin, PI3K/mTOR）信号通路的传导，并且联合应用ML385抑制剂和PI3K抑制剂会增强肺鳞癌的生长抑制功能，提示可以通过靶向NRF2进行干预治疗。

### 3.3 基于LCOs的药物敏感性测试（drug sensitivity testing, DST）

目前所用的临床前模型尚不能准确概括体内药物疗效和细胞毒性导致药物开发效率较低。类器官DST技术是一种基于患者肿瘤组织在体外构建“微型替身”的个性化医疗工具^[50]^，通过模拟药物反应可预测个体对治疗方案的疗效，从而指导治疗决策。因此对于经指南推荐方案治疗效果仍不理想的肺癌患者，在遵守相应的法律法规并征得其家属知情同意的情况下，可建议患者进行肿瘤类器官培养和DST检测，根据患者肿瘤特性制定个体化治疗方案。

科学家已经成功利用类器官DST技术在食管癌、肝癌、肺癌、胰腺癌等多种恶性肿瘤中展现出药物疗效预测潜力^[51-54]^。但目前DST的临床应用尚面临较多局限，主要包括高质量的样本难以获取、TME的体外重建存在难度、检测周期与患者的用药时间存在冲突等；并且目前已成功发表的LCOs模型药敏相关研究多以小样本、单中心回顾性队列为主^[55]^。未来研究的重点是将基于类器官的DST检测结果与患者的临床结局相关联，针对多中心患者开展更广泛的前瞻性队列研究，以证明该检测技术在指导治疗决策和改善患者预后方面的实用性。Zhu等^[56]^开发了一个动态微生理系统芯片平台，并据此开发了一个高通量肺癌球体模型和肠-肝-心-肺癌微生理系统对四种抗肺癌药物进行平行测试，证明了该平台的可行性。通过此平台药物分子被吸收到目标病灶还可以评估真实的药理作用，可进一步应用于疾病建模和药物开发。基于类器官明场识别的治疗筛选（Organoid Brightfield Identification-based Therapy Screening, OrBITS）平台是一种高通量兼容和自动化活细胞图像分析软件。Deben等^[57]^利用此平台对LCOs进行药物筛选，并用金标准ATP测定CellTiter-Glo 3D进行验证，确定OrBITS成像分析在确定半数抑制浓度（50% inhibitory concentration, IC_50_）、细胞活力方面是稳定的，而且可以通过使用标准化药物反应指标和荧光细胞死亡标志物深入了解抗癌药物机制。提示其作为一种可扩展的高通量技术，可以有效促进PDOs在药物开发、治疗筛选和个性化医疗中的应用。

肿瘤类器官生物样本库是经标准化采集、体外培养扩展并长期冷冻保存的肿瘤类器官模型活体样本库，冻存标本复苏后仍能保持原有的生物学行为和遗传特性，为肿瘤机制研究、新药筛选提供了高保真的体外测试平台。Wensink等^[58]^、Dayton等^[59]^均成功建立了PDOs诊治平台，并进行了基因族谱分析和药物筛选，处于不同治疗阶段或治疗方式的肿瘤患者癌组织均可以从肿瘤类器官诊治平台中获益。但广泛的应用和发展也标志着活体类器官生物样本库的建立需要更高的技术手段和更严格的技术标准，这也始终是困扰样本库大规模构建的根本原因（图2）。

**图 2:**
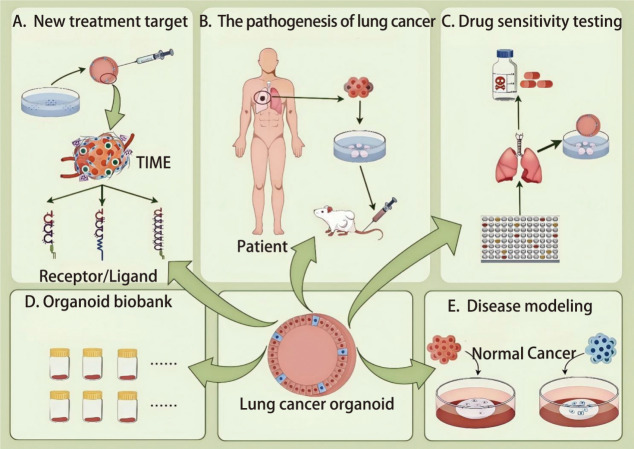
LCO在肺癌精准诊疗中的应用

## 4 小结与展望

当前肺癌研究领域正经历着从“传统单科经验式诊疗”向“个体精准诊疗模式”的重大转变。3D类器官培养技术具备自发性组织特征，并能保留亲本遗传因素和肿瘤异质性，已逐渐成为癌症研究的理想工具。在这里我们对其在肺癌精准诊疗中的应用和进展进行了系统综述。除此之外，其在肿瘤-免疫共培养技术的应用、肿瘤类器官生物样本库的建立以及血管化类器官技术的突破等方面也展现出极其重要的临床转化价值。

但其在临床转化应用仍存在诸多挑战，主要包括：（1）LCOs培养构建过程中组织不新鲜、细胞量不足、缺乏特异性细胞因子、细菌污染等问题都会对LCOs模型的成功培养产生影响。（2）现有类器官模型大多缺乏脉管系统、免疫细胞及神经、血管在内的特殊环境，无法完全复刻肿瘤在体内TIME中的生长、浸润情况，结果缺乏真实性。（3）同一原发病灶的不同区域之间存在着遗传、表型亚克隆差异，靶向药物持续干预下肿瘤也会发生不同程度的扩增、演化，最终形成获得性耐药。（4）肿瘤类器官生物样本库培养成功率低、保存时间短、技术标准严格也始终是困扰其实现临床转化的重要难题。类器官培养技术的快速发展已经在肺癌研究领域产生了重要的影响。随着越来越多的研究人员的参与、构建工艺的不断改进和应用范围的不断扩大，我们相信类器官技术必将改写肺癌当前的诊疗格局，显著提高患者的生存质量和生存期，并有望成为肺癌精准诊疗时代的核心工具。
